# Last Licensed Non-Classical Peptide Presenter HLA-F: Its Occurrence, Immunoediting Significance, and Possible Therapeutic Implications in Renal Cell Carcinoma

**DOI:** 10.3390/ijms27115069

**Published:** 2026-06-03

**Authors:** Libuše Janků, Michal Fedorko, Marek Kašík, Táňa Macháčková, Leoš Křen

**Affiliations:** 1 Transfusion and Tissue Department, University Hospital Brno, 625 00 Brno, Czech Republic; janku.libuse@fnbrno.cz; 2 Department of Laboratory Medicine, Faculty of Medicine, Masaryk University, 625 00 Brno, Czech Republic; 3 Department of Urology, University Hospital Brno, Faculty of Medicine, Masaryk University, 625 00 Brno, Czech Republic; fedorko.michal@fnbrno.cz (M.F.); kasik.marek@fnbrno.cz (M.K.); 4 Department of Biology, Faculty of Medicine and CEITEC, Masaryk University, 625 00 Brno, Czech Republic; tana.machackova@gmail.com; 5 Department of Pathology, Faculty of Medicine, Masaryk University, 625 00 Brno, Czech Republic; 6 Department of Pathology, University Hospital Brno, 625 00 Brno, Czech Republic

**Keywords:** HLA-F, renal cell carcinoma, non-classical HLA molecules

## Abstract

Among non-classical human leukocyte antigen (HLA) molecules, HLA-F remains the most enigmatic. Its precise functions, including the recently described peptide-presenting capability, are still not fully understood. Renal cell carcinoma (RCC) is characterized by its resistance to radiotherapy and chemotherapy, and it is known for its immunogenicity. The prognostic significance of HLA-F expression in neoplastic cells has not been well defined. We evaluated HLA-F–specific mRNA transcripts in 73 RCC samples and 54 adjacent normal kidney parenchyma samples and statistically correlated the results with several clinicopathological parameters. Our analysis revealed that HLA-F is upregulated in RCC. Interestingly, higher HLA-F expression was associated with prolonged disease-free survival, more favorable pT stage, and lower WHO/ISUP grade. Given the potential for interferon-mediated aberrant activation of non-classical HLA molecules, assessment of HLA-F status could help identify RCC patients who might benefit from tailored neoadjuvant biological or immunological therapies. Overall, HLA-F may serve as a valuable prognostic biomarker in RCC, and further studies are warranted to elucidate its functional and clinical significance.

## 1. Introduction

The human leukocyte antigen (HLA) system is a cluster of more than 200 genes on the short arm of chromosome 6p21.3, of which around 40–50 encode classical and non-classical HLA molecules, making it one of the most gene-dense and polymorphic regions of the human genome [1]. The HLA system plays a central role in immune recognition and surveillance, orchestrating adaptive and innate responses by presenting antigenic peptides to T lymphocytes and modulating the activity of natural killer (NK) cells. Classical HLA class I molecules HLA-A, -B, and -C (class Ia) are highly polymorphic and ubiquitously expressed, ensuring broad peptide presentation to CD8^+^ T cells. In contrast, non-classical HLA class I (class Ib) molecules, including HLA-E, HLA-G, and HLA-F, display limited polymorphism [2,3], restricted tissue distribution, and specialized immunomodulatory functions [4,5,6]. Non-classical HLA molecules were originally thought to be specifically expressed only on extravillous trophoblasts [Figures S1 and S2]—fetal tissue in contact with maternal cells that HLA-A and -B antigens, assuring the protection of fetal semiallogeneic graft from maternal allorecognition [7,8,9]. Among non-classical HLA class I molecules expressed in humans, HLA-F, discovered in 1990, is the least characterized with regards to its expression and/or function(s), including immunoregulatory properties. In addition, information on the clinicopathological significance of HLA-F presence in tumors is limited. Unlike classical HLA class I molecules, HLA-F exhibits a restricted tissue distribution and limited polymorphism and is highly conserved in distantly related non-human primates, suggesting a conserved role [10].

Non-classical HLA molecules play a crucial role in modulating immune responses through interactions with inhibitory and activating receptors on immune effector cells. HLA-F has recently gained attention as a uniquely licensed peptide-presenting molecule with distinct immunological properties [11]. HLA-F can exist in two main conformations: an open conformer (OC), in which the heavy chain is not associated with β2-microglobulin or peptide, and a β2-microglobulin–associated, peptide-bound form (β2m–HLA-F). These distinct conformations enable HLA-F to interact with both inhibitory and activating receptors on immune cells. The open conformer of HLA-F preferentially engages killer immunoglobulin-like receptors (KIRs), including the inhibitory receptors KIR3DL1 and KIR3DL2, as well as the activating receptor KIR2DS4, expressed predominantly on NK cells and subsets of T cells. In contrast, the β2m–HLA-F complex interacts with inhibitory leukocyte immunoglobulin-like receptors, primarily LILRB1 (ILT2) and LILRB2 (ILT4), thereby contributing to immune inhibitory signaling in the tumor microenvironment [12]. These interactions enable HLA-F to modulate innate and adaptive immune effector functions, influencing NK cell activation, cytokine production, and cytotoxic responses [13,14]. Dulberger at al demonstrated that HLA-F is capable of presenting endogenous peptides, contrary to previous assumptions that it exists primarily as an empty molecule. The authors further showed that HLA-F binds unusually long peptides via an open-ended binding groove and that peptide loading modulates its interactions with NK cell receptors, thereby influencing immune regulation [15].

HLA-F expression can be upregulated in various malignancies, implicating it in tumor immune escape and immunoediting [16,17,18]. The most studied non-classical HLA molecules in solid tumors is HLA-G, and some data regarding HLA-E also have been published [19,20,21]. Emerging evidence suggests that HLA-F expression is altered in various malignancies and may contribute to tumor immune escape and the shaping of the tumor microenvironment. The impact of HLA-F expression in cancer is a matter of controversy. Although most studies associate high HLA-F expression with adverse prognosis in several solid tumors [22,23,24], some reports have found no significant correlation between HLA-F expression and survival or clinicopathological parameters in certain cancer types [25]. In triple-negative breast cancer, the combined low expression of HLA-F and CD56 was associated with prolonged relapse-free survival and longer time to recurrence, highlighting the importance of immune context in disease progression [26]. Despite emerging evidence on the immunological functions of HLA-F, its role in oncology remains poorly understood. Current data are limited, and the implications of HLA-F expression in tumor progression and patient prognosis are largely unexplored. Further studies are therefore essential to elucidate its clinical significance and potential as a therapeutic target.

Renal cell carcinoma (RCC), as shown in Figure S3, is characterized by its resistance to radiotherapy and/or chemotherapy. On the other hand, it is one of several immunogenic tumors. Data on the precise role of HLA-F in RCC biology and its potential as a prognostic biomarker or therapeutic target are currently very limited or entirely lacking in large cohort studies. The aim of this study was to investigate the detection of primary HLA-F mRNA transcripts in RCC and to evaluate the impact of HLA-F expression on clinical and pathological parameters. Our goal was to clarify the potential prognostic significance of HLA-F expression in RCC and its relationship with tumor progression.

## 2. Results

### 2.1. Patients Cohorts

Frozen specimens from 73 RCC patients were included in this study. All cases represented consecutive primary diagnoses and were obtained from the Tissue Bank of the Masaryk Memorial Cancer Institute (Brno, Czech Republic). Patients’ ages at surgery ranged from 35 to 82 years, with a median of 63 years. Histological diagnoses, including WHO/ISUP grading, were established according to the latest WHO classification guidelines [27]. Cases were selected based on tissue availability and were not stratified by any known preoperative or pathological prognostic factors. Clinical follow-up data, including annually assessed survival, were available for all patients. No patient received neoadjuvant therapy, including biological or immunotherapy. The median follow-up was 24.5 months. Clinical characteristics of the patient cohort are summarized in Table 1.

### 2.2. Differential Expression of HLA-F mRNA in RCC and Non-Neoplastic Renal Parenchyma

**Figure 1:**
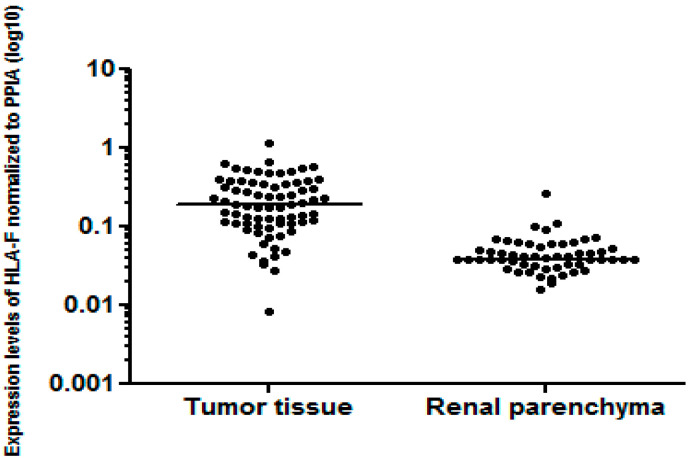
HLA-F expression in RCC tissues and non-neoplastic renal parenchyma. PPIA—peptidyl-prolyl isomerase A.

**Figure 2:**
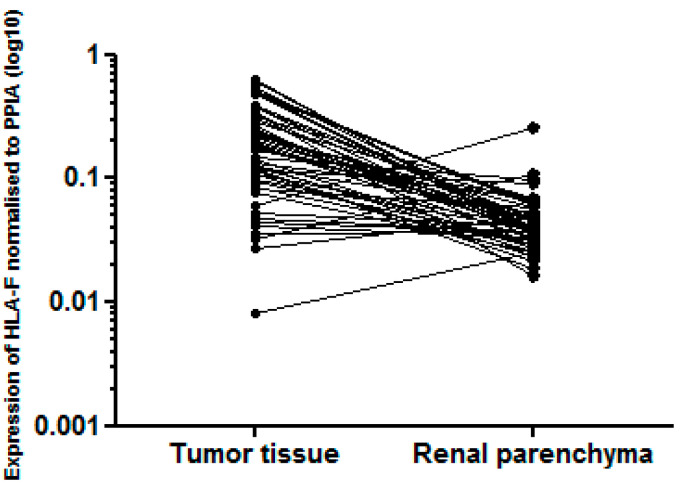
HLA-F expression in RCC tissues and non-neoplastic renal parenchyma based on paired samples.

The presented data indicate that HLA-F–specific mRNA transcripts were detectable in both RCC tumor tissues (n = 73) and non-neoplastic renal parenchyma (n = 54). A significantly different expression of HLA-F between RCC tissues and non-neoplastic renal parenchyma was observed (*p* < 0.0001), as shown in Table 2 and Figure 1. Using the Wilcoxon signed-rank test, significantly higher HLA-F expression was detected in tumor tissues compared with paired non-neoplastic samples (Figure 2; *p* < 0.0001, ***).

### 2.3. Prognostic Impact of HLA-F Expression

**Figure 3:**
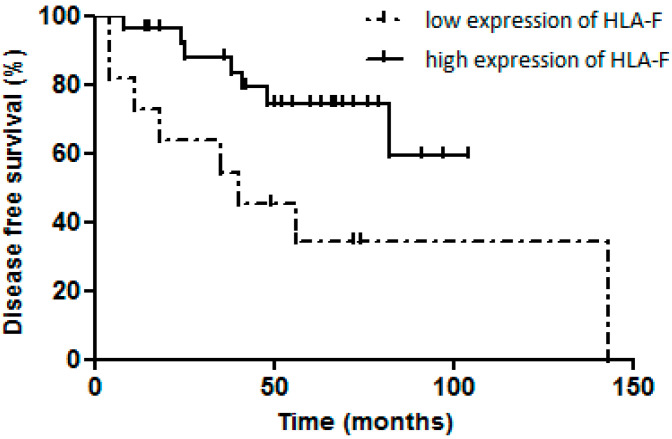
Disease-free survival according to HLA-F expression (n = 73).

**Figure 4:**
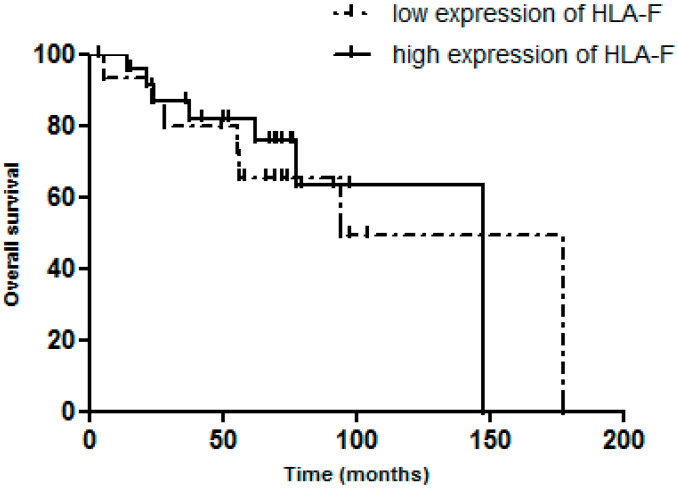
Overall survival after surgery according to HLA-F expression.

Disease-free survival of RCC patients (n = 73) stratified according to HLA-F expression is shown in Figure 3. The analysis revealed a significant positive association between HLA-F expression and time to progression (*p* = 0.0226, log-rank test). Overall survival after surgery according to HLA-F expression is depicted in Figure 4. No significant association between HLA-F expression levels and overall survival was observed (cut-off value = 0.1849; *p* = 0.7993, log-rank test).

**Figure 5:**
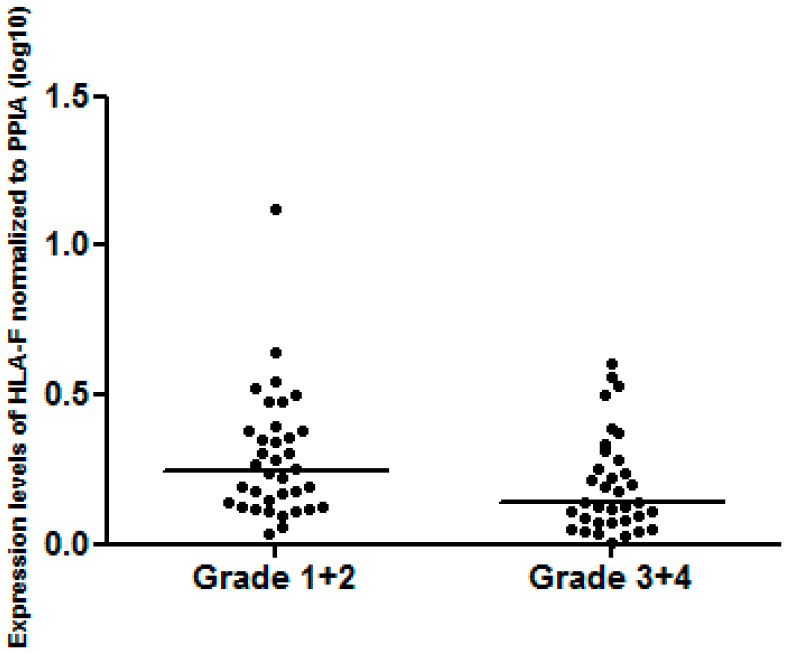
HLA-F expression and WHO/ISUP grade.

**Figure 6:**
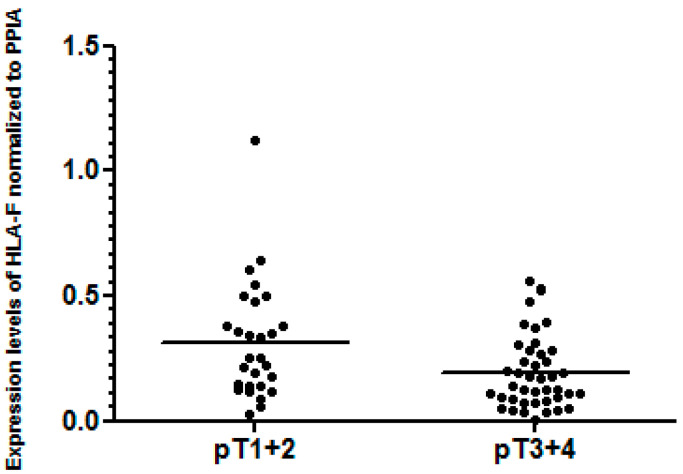
HLA-F expression and pT stage.

The relationship between HLA-F expression and WHO/ISUP grade is presented in Figure 5. Lower HLA-F expression was significantly associated with higher WHO/ISUP grade, indicating a negative correlation between HLA-F expression and tumor grade (*p* = 0.0218, Mann–Whitney U test). Furthermore, HLA-F expression was significantly associated with tumor pT stage. Higher HLA-F expression was observed in lower pT stages, with significant differences between low and advanced pT categories (Figure 6).

## 3. Discussion

Non-classical immunomodulatory molecules HLA-E, -F, and -G were originally thought to be selectively expressed on extravillous trophoblasts. This observation suggested a physiological role for these “pregnancy-associated” molecules in the development of maternal tolerance to the semi-allogeneic fetus, representing one of the mechanisms preventing embryo demise in utero (“sentinels of pregnancy”) [7,8,9].

It has been demonstrated that HLA-F can exist in distinct conformations, including an open conformer and a β2-microglobulin–associated, peptide-bound form, which enable interactions with inhibitory receptors such as ILT2 and ILT4, as well as with certain KIRs, suggesting a mechanism by which HLA-F may contribute to immune tolerance [11,12,13,14,15].

Regarding the expression and function of HLA-F in neoplastic processes, available data are scarce and often contradictory. HLA-F protein expression has been identified in several human malignancies, with conflicting results concerning its prognostic significance. Published studies on HLA-F expression in solid tumors reveal a heterogeneous picture with respect to both expression patterns and clinical relevance. Most reports associate HLA-F positivity with poorer prognosis across different tumor histotypes. High HLA-F expression has been linked to unfavorable outcomes in esophageal squamous cell carcinoma [28], non-small-cell lung carcinoma [29], and hepatocellular carcinoma [24]. In contrast, one study reported a favorable prognostic impact of elevated soluble plasma HLA-F levels in metastatic neuroblastoma, showing improved event-free and overall survival [30]. Very recently, a significant interaction between HLA-F and CD56 in relation to disease-free survival and time to recurrence was reported in triple-negative breast cancer patients [26]. Collectively, these findings indicate that although HLA-F is frequently expressed in various solid tumors, its prognostic significance appears to be tumor-type specific.

In the present study, we demonstrate that HLA-F expression is detectable in both normal and malignant renal tissues and is significantly higher in RCC tumor tissue compared with adjacent non-tumor renal parenchyma. Moreover, HLA-F expression was negatively associated with tumor aggressiveness, as reflected by WHO/ISUP grade and pT stage, and positively correlated with longer relapse-free survival. However, no significant association was observed between HLA-F expression and overall survival in our cohort. These findings suggest that HLA-F may be involved in RCC progression and may play a role in early tumor development or immune interactions that delays disease progression but does not necessarily influence long-term survival. Importantly, our results further indicate a positive association between high levels of HLA-F–specific mRNA transcripts and prolonged relapse-free survival in RCC patients.

Aberrant induction of HLA-F expression in response to interferon-γ stimulation has been previously described [31]. We speculate that interferon-γ–mediated upregulation of HLA-F in initially HLA-F–negative RCC tumors may represent one of the mechanisms contributing to the therapeutic efficacy of interferon-based immunotherapy in selected patients.

In summary, we demonstrate for the first time a positive prognostic significance of HLA-F transcripts in RCC. Furthermore, we show for the first time that increased HLA-F transcript levels are associated with more favorable pT stage and lower WHO/ISUP grade. These findings support the potential of HLA-F as a prognostic biomarker in RCC. Further studies are warranted to elucidate the immunomodulatory mechanisms of HLA-F in the context of tumor immune surveillance and activation–inhibition balance. Characterization of HLA-F status may thus contribute to improved risk stratification and monitoring of RCC patients.

## 4. Materials and Methods

### 4.1. Tissue Sample Preparation and RNA Purification

A total of 127 tissue samples were collected prior to the initiation of any treatment from surgically resected specimens under the supervision of an experienced pathologist. These included 73 samples from primary cell renal carcinoma (RCC) tumors obtained from 73 patients and 54 samples of adjacent non-tumoral renal parenchyma obtained from 54 patients (the remaining 19 patients did not have non-tumoral parenchyma attached to the tumors because only enucleation/tight resection was performed). The inclusion criterion was a minimum of 70% tumor cell content in tumor specimens, confirmed by frozen-section evaluation of the sampled tissue. All specimens were snap-frozen in liquid nitrogen immediately after resection and subsequently stored at −80 °C until RNA extraction. Tissue samples were homogenized under sterile conditions using a MagNA Lyser Instrument (Roche Applied Science, Mannheim, Germany). Total RNA was isolated using the mirVana™ miRNA Isolation Kit (Ambion, Austin, TX, USA) according to the manufacturer’s instructions. RNA concentration and purity were assessed with UV spectrophotometry using a NanoDrop ND-1000 spectrophotometer (Thermo Scientific, Wilmington, DE, USA), with acceptance criteria of A260/A280 > 2.0 and A260/A230 > 1.8.

### 4.2. Reverse Transcription

Complementary DNA (cDNA) was synthesized from 1 μg of total RNA using the High-Capacity cDNA Reverse Transcription Kit (Applied Biosystems, Foster City, CA, USA) according to the manufacturer’s instructions. Each reverse transcription reaction (20 μL total volume) contained 10 μL of diluted RNA sample (100 ng/μL), 2 μL of 10× RT buffer, 0.8 μL of 25× dNTP mix (100 mM), 2 μL of 10× random primers, 1 μL of MultiScribe™ Reverse Transcriptase, and 4.2 μL of DEPC-treated water. The reaction mixtures were incubated for 10 min at 25 °C, followed by 120 min at 37 °C and 5 min at 85 °C to inactivate the enzyme, and then held at 4 °C using a T100™ Thermal Cycler (Bio-Rad, Hercules, CA, USA).

### 4.3. Real-Time qPCR Analysis

Quantitative real-time PCR (RT-qPCR) was performed using the Applied Biosystems 7500 Sequence Detection System (Applied Biosystems, Foster City, CA, USA) according to the manufacturer’s recommendations. Each PCR reaction (20 μL total volume) contained 1 μL of reverse transcription product, 10 μL of TaqMan^®^ Gene Expression Master Mix, 8 μL of DEPC-treated water, and 1 μL of the TaqMan^®^ Gene Expression Assay for HLA-F (Assay ID: Hs04193807_g1; Applied Biosystems, Foster City, CA, USA).

Amplification was carried out in 96-well optical plates under the following cycling conditions: 50 °C for 2 min, 95 °C for 10 min, followed by 40 cycles of 95 °C for 15 s and 60 °C for 1 min. Ct values were determined using default threshold settings. All samples were analyzed in duplicate, and inter-plate controls (IPC) as well as non-template controls (NTC) were included in each run. Mean Ct values and standard deviations were calculated. The expression levels of HLA-F mRNA were normalized to the endogenous reference gene PPIA (peptidylprolyl isomerase A) using the ΔCt method. Relative gene expression was calculated using the 2^−ΔCt^ method for paired tumor vs. adjacent non-tumoral tissue comparisons. The stability of PPIA expression across tumor and non-tumor renal tissues was verified prior to normalization.

### 4.4. Data Normalization and Statistical Analysis

Threshold cycle (Ct) values were calculated using SDS software version 2.0.1 (Applied Biosystems, Foster City, CA, USA) with default threshold settings. To correct for inter-plate variability, Ct values of target genes were adjusted using an inter-plate control (IPC) according to the following formula:

Ct(corrected) = Ct(mean of duplicates) − Ct(IPC (plate)) + mean Ct(IPC (all plates)).

Expression levels were normalized to the endogenous reference gene PPIA (Assay ID: Hs99999904_m1; Applied Biosystems, Foster City, CA, USA) using the ΔCt method. Relative gene expression was calculated as follows:

2^−ΔCt^ = 2^−(Ct(target) − Ct(PPIA))^

Statistical analyses were performed using GraphPad Prism 9 (GraphPad Software, San Diego, CA, USA). Data were presented as mean ± standard deviation (SD). Differences between two groups were analyzed using the Mann–Whitney U test for unpaired samples and the Wilcoxon signed-rank test for paired samples. Correlations between gene expression and clinicopathological parameters were assessed using Spearman’s rank correlation coefficient. Survival analyses were performed using the Kaplan–Meier method, and differences were evaluated using the log-rank test. A *p*-value < 0.05 was considered statistically significant.

### 4.5. Presence and Prognostic Impact of HLA-F

HLA-F–specific mRNA transcripts were detectable in both tumor tissues of RCC samples (n = 73) and adjacent non-neoplastic renal parenchyma (n = 54). Significantly higher expression of HLA-F in RCC tissues compared to non-neoplastic renal parenchyma was observed (*p* < 0.0001; Table 2, Figure 1). In paired samples, analysis using the Wilcoxon signed-rank test confirmed significantly elevated HLA-F expression in tumor tissues relative to corresponding non-neoplastic tissues (*p* < 0.0001; Figure 2).

Relapse-free survival of RCC patients (n = 73) stratified by HLA-F expression is shown in Figure 3. A positive correlation between higher HLA-F expression and longer time to progression was observed (*p* = 0.0226; *, log-rank test). Overall survival after surgery according to HLA-F expression is presented in Figure 4. No significant association between HLA-F expression and overall survival was found (cut-off = 0.1849; *p* = 0.7993; log-rank test). Analysis of HLA-F expression in relation to WHO/ISUP grade demonstrated a negative correlation between HLA-F levels and higher nuclear grades (*p* = 0.0218; *, Mann–Whitney test; Figure 5). Similarly, HLA-F expression was associated with tumor pT stage. Significant differences were observed, with higher expression detected in tumors of lower pT stage (Figure 6).

## 5. Conclusions

The role of the immunomodulatory non-classical molecule HLA-F in the development and clinical course of RCC remains incompletely characterized. In this study, we demonstrated that HLA-F mRNA transcripts are present in both normal kidney tissue and RCC and that higher transcript levels were unexpectedly associated with prolonged disease-free survival, more favorable pT stage, and lower WHO/ISUP grade. Given the reported regulation of non-classical HLA molecules by interferons, HLA-F expression may also be relevant in the context of immunomodulatory treatment strategies in RCC. However, the clinical implications of these observations remain to be established. Overall, our findings suggest that HLA-F could represent a potential prognostic biomarker in RCC, although further validation in larger and independent cohorts is warranted.

## Figures and Tables

**Table 1:** Clinical and pathological characteristics of the RCC patient cohort (n = 73).

Characteristic	N (%) or Range	Median
Age at diagnosis (years)	35–82	63
Gender	Male: 47 (64.4%) Female: 26 (35.6%)	-
Histology	ccRCC: 63 (86.3%) Papillary: 5 (6.8%) Chromophobe: 1 (1.4%) SAR: 1 (1.4%) URO: 1 (1.4%) Not specified: 2 (2.7%)	-
T category	1: 20 (27.4%) 2: 8 (11.0%) 3: 40 (54.8%) 4: 2 (2.7%) Not specified: 3 (4.1%)	-
WHO/ISUP grade	1: 8 (11.0%) 2: 28 (38.4%) 3: 23 (31.5%) 4: 12 (16.4%) Not specified: 2 (2.7%)	-
Disease-free survival (months)	Range: 3–143 Missing: 33	49.5
Overall survival (months)	Range: 0–177 Missing: 1	24.5
Metastasis	Yes: 36 (49.3%) No: 33 (45.2%) Missing: 4 (5.5%)	-

ccRCC—clear cell renal cell carcinoma; SAR—sarcomatoid renal cell carcinoma; URO—urothelial renal cell carcinoma.

**Table 2:** HLA-F mRNA expression in RCC and adjacent non-neoplastic renal tissue.

Non-Paired Samples	RCC (n = 73)	Adjacent Non-Tumor Renal Parenchyma (n = 54)	*p*-Value
25th percentile	0.1087	0.0320	
50th percentile (median)	0.1892	0.0383	<0.0001
75th percentile	0.3484	0.0545	
Paired samples	RCC (n = 54)	Adjacent Non-Tumor Renal Parenchyma (n = 54)	*p*-Value
25th percentile	0.0969	0.0320	
50th percentile (median)	0.1773	0.0383	<0.0001
75th percentile	0.3484	0.0545	

## Data Availability

Data supporting the reported results can be found.

## Supplementary Materials

The following supporting information can be downloaded at: https://www.mdpi.com/article/10.3390/ijms27115069/s1.

## Abbreviations

The following abbreviations are used in this manuscript:

HLA	Human leukocyte antigen
NK	Natural killer
OC	Open conformer
β2m	β2-Microglobulin
KIR	Killer immunoglobulin-like receptor
LILRB1	Leukocyte immunoglobulin-like receptor B1
ILT2	Immunoglobulin-like transcript 2
LILRB2	Leukocyte immunoglobulin-like receptor B2
ILT4	Immunoglobulin-like transcript 4
RCC	Renal cell carcinoma
mRNA	Messenger RNA
PPIA	Peptidylprolyl isomerase A
