# Supplementary figures and images for: Last Licensed Non-Classical Peptide Presenter HLA-F: Its Occurrence, Immunoediting Significance, and Possible Therapeutic Implications in Renal Cell Carcinoma
Source: Int J Mol Sci. 2026 Jun 3;27(11):5069. doi: 10.3390/ijms27115069 (PMC13257176; doi:10.3390/ijms27115069)

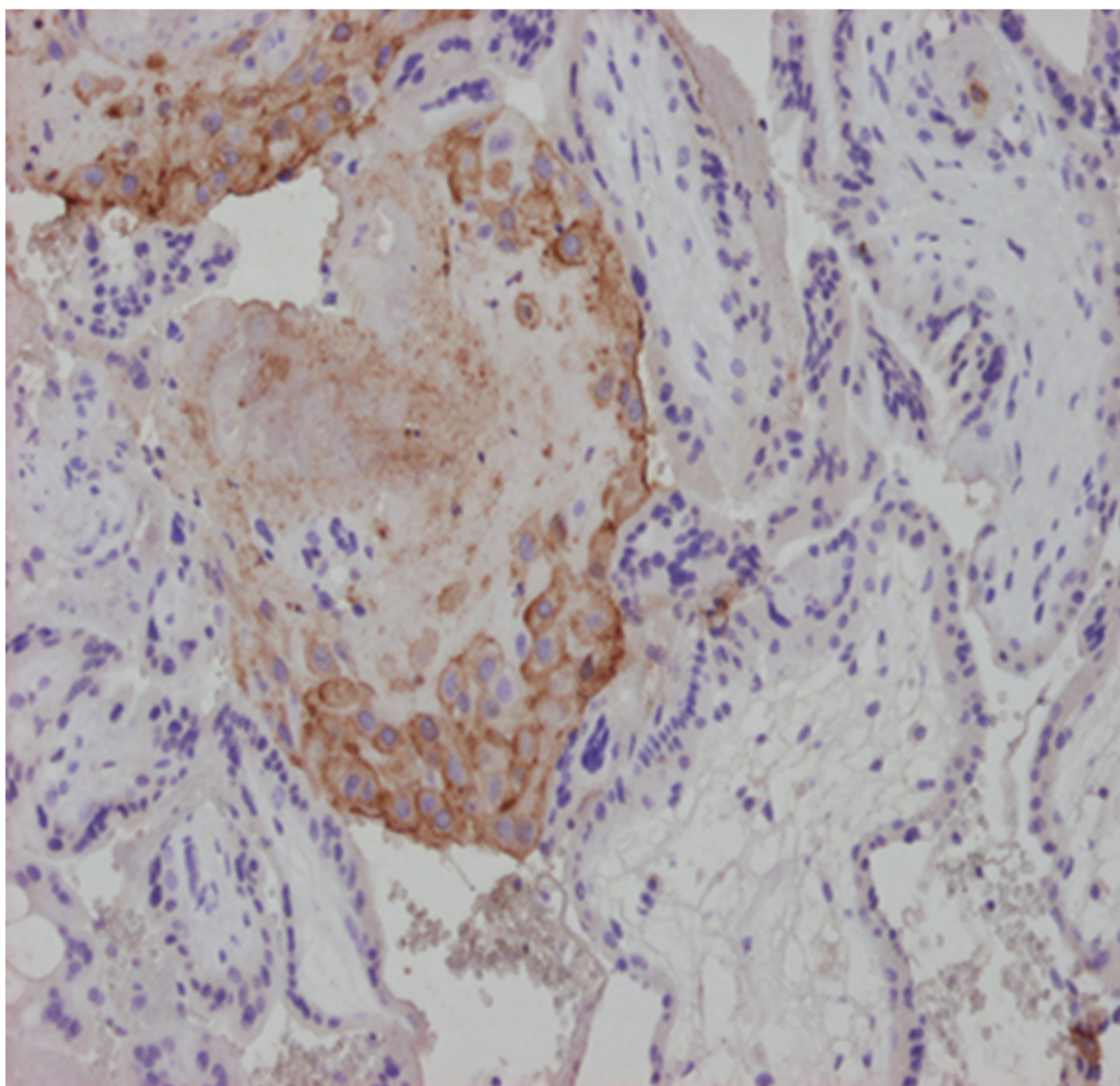

Figure S2

Supplement: Supplementary file 1 [file ijms-27-05069-s001.zip › Figure S2.pdf]

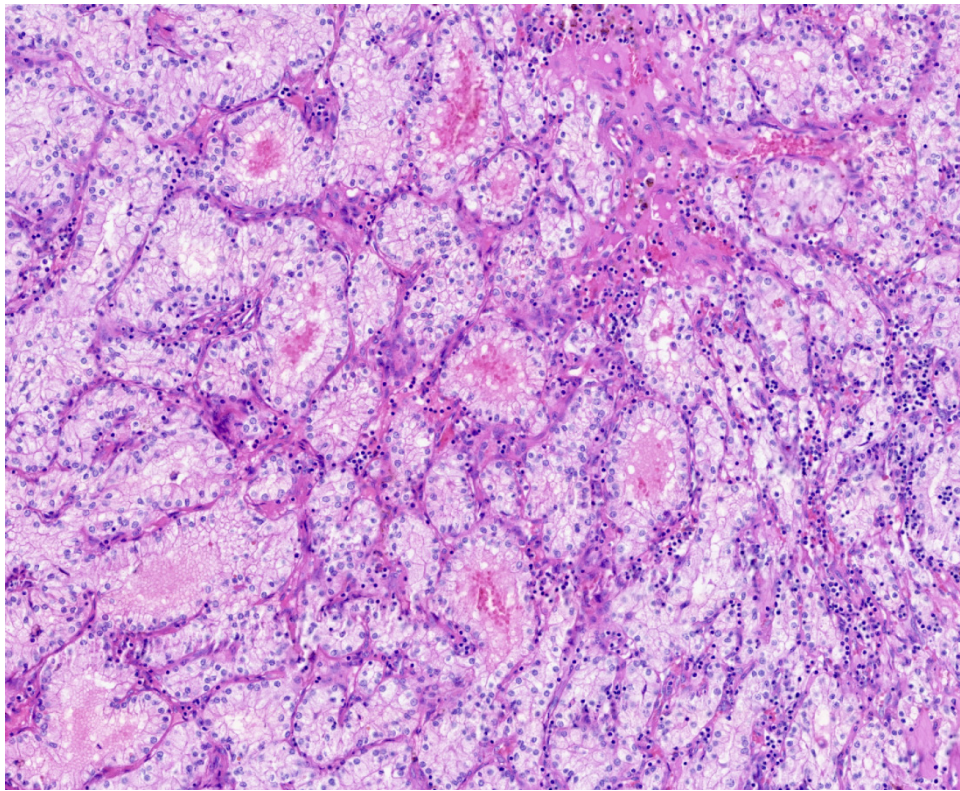

Figure S3

Supplement: Supplementary file 1 [file ijms-27-05069-s001.zip › Figure S3.pdf]

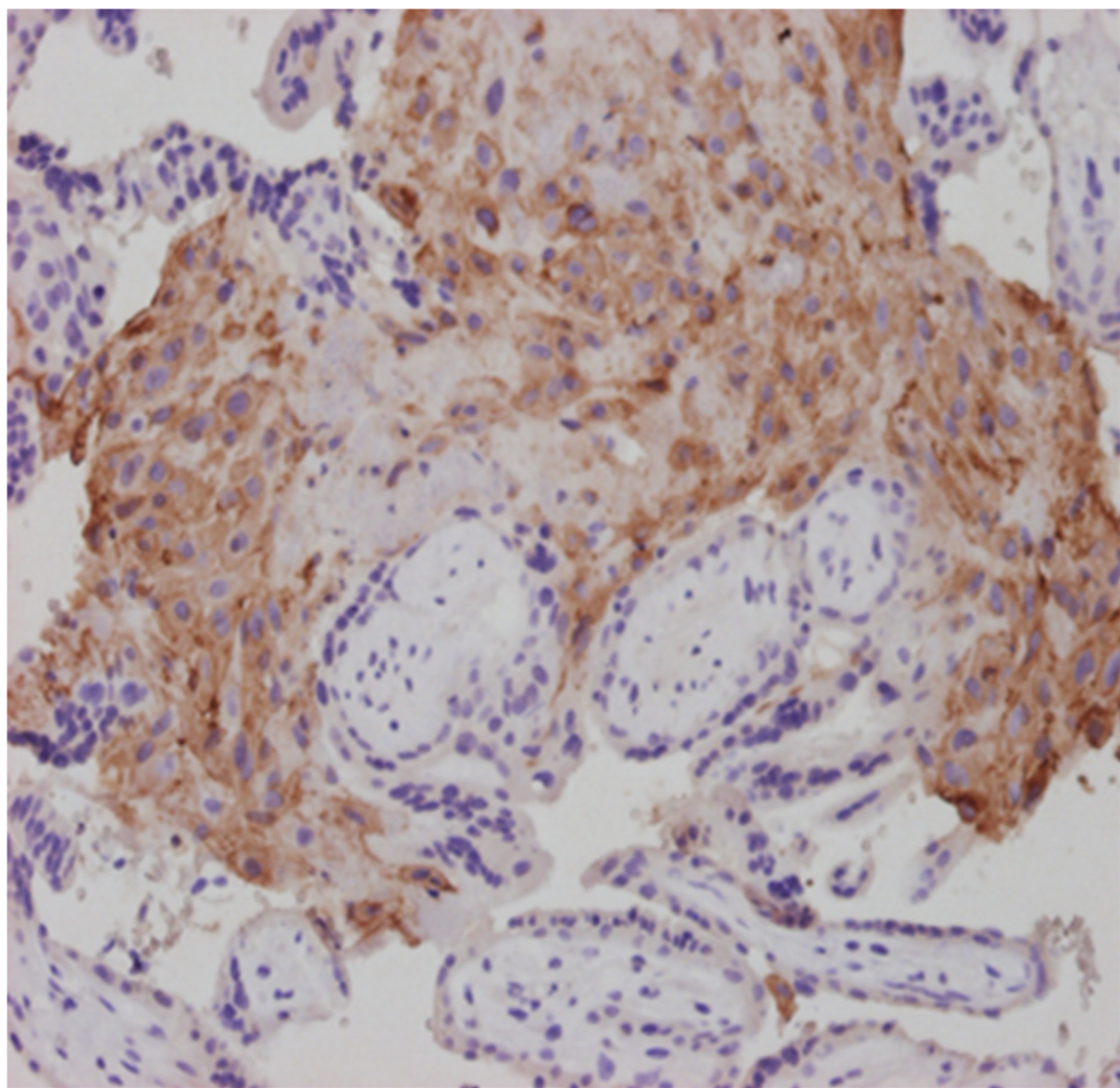

Figure S1

Supplement: Supplementary file 1 [file ijms-27-05069-s001.zip › Figure S1.pdf]
